# Same game, different worlds? General conditions, perceived stress, and associations between stress and past season injuries in elite female and male ice hockey players

**DOI:** 10.1186/s13102-024-00862-0

**Published:** 2024-03-20

**Authors:** Tobias Wörner, Stefan Kauppinen, Frida Eek

**Affiliations:** 1 Department of Health Sciences, Lund University, Box 157, Lund, 22100 Sweden; 2 Stockholm Sports Trauma Research Center, Department of Molecular Medicine and Surgery, Karolinska Institutet, Stockholm, Sweden

**Keywords:** Gender equity, Hockey, Life stress, Psychological stress, Athletic injuries

## Abstract

**Background:**

Ice hockey is played by women and men but the arena they play in may differ substantially. Potential differences in general conditions to play the sport may be associated to perceived stress, which has shown to be related to athletic injury in other sports. Therefore, this study aimed to describe and compare general conditions for playing ice hockey, stress levels, and the association between perceived stress and injury occurrence among elite female and male players.

**Methods:**

Prior to the 2022–2023 season all female and male players from the top ice hockey leagues in Sweden were invited to an online survey. Players provided information about their general conditions for playing ice hockey and reported perceived stress during the previous season on the Perceived Stress Scale (PSS-10; sum score range 0–40) and current stress on the Single Item Stress Question (SISQ; scale range 1–5). Injuries during the previous season were self-reported on a modified version of the Oslo Sports Trauma Research Center – Overuse Injury Questionnaire.

**Results:**

We received responses from 360 players (170 females and 190 males). Female players more frequently reported additional occupations besides ice hockey and less medical support during games and practices than male players (*p* < .001). General stress levels were significantly higher among female players (mean PSS score [SD]: 17.4 [5.6] vs 14.1 [5.6], *p* < 0.001; SISQ median [IQR]: 3 [2–3] vs 2 [1–3], *p* < 0.001). There was a statistically significant but weak correlation between past season injury and perceived stress (PSS score: rho 0.29; SISQ: rho 0.24). This correlation was stronger among males than females (PSS score: 0.38 vs 0.162; SISQ: 0.29 vs 0.16, *p*’s < 0.05). Players with substantial injuries during previous season had higher previous and current stress levels than players without injury, a difference that was largest and statistically significant only among male players.

**Conclusions:**

General conditions for playing elite ice hockey are inequal for female and male athletes. Stress levels of elite ice hockey players were comparable to the general population. Experience of severe injuries during the previous season was associated to higher levels of perceived stress. This association is stronger among male players, which may be due to greater economic dependency.

## Background

“When I was a kid, I used to dream about becoming an ice hockey star. I stopped dreaming when I grew older because the dream is impossible”. This paraphrased quote comes from a qualitative study interviewing female ice hockey players on their material and structural conditions within their sport in Sweden [1]. Since 2008, when this study was published, the number of girls and women playing hockey in Sweden has increased by 100%. Today, one in every 10 registered ice hockey player in Sweden is female [2]. Although they play the same sport as their male counterparts, the conditions for female hockey players are likely to differ immensely.

Widening the scope from Sweden to a global scale, the Olympic games are a prime example of the slow but ongoing process towards gender equality in sports. Throughout the past 123 years, the number of female participants in the games has risen from 2% in the 1900 Paris to 48% in the 2022 Tokyo games [3]. Apart from female representation during the competitions, the visibility of female athletes was increased when the world saw one female and one male athlete carrying the flag of 91% of participating nations in the 2020 Tokyo [3]. In contrast to this, the coverage of female sports in Sweden is reported to be as low as 11% of all sports shown on national television [4]. Professional sport is a business and media coverage is one of the decisive factors for distribution of resources. Hence, the striking inequality in media coverage is a strong indicator for existing differences in general conditions between male and female athletes.

While some elite male ice hockey players receive generous financial compensations to be a full-time athlete, female players rarely receive the same compensations, and it is likely that female players must juggle hockey with additional occupations to meet the demands for everyday life. According to the demand-control model, an imbalance between perceived demands and resources/control may lead to stress, with both psychological and physical responses [5]. The situation may be further amplified by low social support [6]. This type of stress, related to an imbalance between job demands and control has over longer periods of time been related to health problems such as type 2 diabetes, coronary heart disease, and work absence due to mental health problems [7–9]. Sufficient time for recovery may, however, reduce the risk for chronic stress and related health problems [10]. For decades it has been hypothesized that stress also is linked to athletic injury [11] and recent systematic reviews and meta-analyses strengthen this theory by finding it to be associated with the risk for acute and overuse injuries [12, 13]. Conversely, injured athletes have found to experience negative psychological and physiological stress responses [14]. While female and male athletes may play the same sport, they play the sport in different contextual arenas. Yet, these contextual differences, shaping the general conditions to be an elite athlete in society, have yet to be compared between elite female and male ice hockey players. Although it is plausible that the differences between the general conditions for male and female elite athletes lead to different levels of stress, the potential association between perceived stress and athletic injury has neither been explored nor compared in female and male elite ice hockey.

In this study we aimed to describe and compare general playing conditions, perceived stress, and association between stress levels and injury occurrence among elite female and male ice hockey players. Furthermore, potential associations between perceived stress and past season injuries were investigated among elite female and male ice hockey players.

## Methods

In this cross-sectional study, we invited players from the highest ice hockey leagues for females [Swedish Women’s Hockey League (SWHL)] and men (Swedish Hockey League [SHL], HockeyAllsveskan [HA]) in Sweden to respond to an online survey. SWHL players participate in another prospective study and provided separate informed consent. Players from the SHL and HA provided their informed consent anonymously before answering the online survey. The Swedish ethics review authority approved the study protocol (Dnr 2022–02668-01). We wrote the manuscript with consideration to the Strengthening the Reporting of Observational Studies in epidemiology (STROBE) guidelines [15].

### Participants and recruitment

Ice hockey players in the SWHL (10 teams) as well as SHL (14 teams) and HA (14 teams) were invited to participate in the study. SWHL players were recruited within an ongoing prospective study investigating injuries and health problems in the league. The SWHL management provided us with contact information for all players who received direct e-mail invitations with two reminders between end of August and beginning of September 2022. Hence, the sampling frame for female players included the total target population at the time of recruitment. For male players we relied on voluntary response sampling by indirectly inviting them via official league managements and club’s medical teams between September and October 2022. They received a public link via email and were provided with a QR-code for the survey in their locker rooms.

### Data collection

Data were collected online through respondent-surveys for female players and anonymous surveys for male players. Players provided demographic information and reported general conditions for playing ice hockey (occupation besides ice hockey, family situation, medical support), perceived stress and information regarding injuries during the previous season.

#### Description of general conditions for playing ice hockey

We collected information about general conditions for playing ice hockey by asking questions about occupational obligations besides playing ice hockey, living- and family conditions, as well as available medical support. Players reported whether their primary occupation is playing ice hockey or if they have other occupational duties such as work or studies. Players also reported if they live by themselves or together with their parents, partners, or children. Finally, we asked a series of questions about the medical support available to players during games, matches, and outside of the sport.

#### Measurement of perceived stress

Perceived Stress Scale (PSS-10) was used to assess perceived stress during the previous season, and current level of perceived stress was assessed by single item stress question (SISQ). PSS-10 consists of 10 items that measure the degree to which life is/has been perceived as unpredictable, uncontrollable, and overloading. These 10 items are answered on a 5-point Likert scale (0–4) and summarized as a total score ranging from 0 (no perceived stress) to 40 (highest degree of perceived stress). PSS-10 is a valid and reliable measure [16] that has been validated in the Swedish language [17]. SISQ, a single item formulated *“Stress means a situation in which a person feels tense, restless, nervous, or anxious or is unable to sleep at night because his/her mind is troubled all the time. Do you feel this kind of stress these days?”* is a valid way to measure current stress in survey-research [18–20]. SISQ is answered on a 5-point Likert scale ranging from 1 (no perceived stress) to 5 (highest degree of perceived stress).

#### Measurement of past season injury

An injury was defined as self-reported “injury or pain” during the past season in one of the following 8 anatomical areas: Head/face/neck, shoulder, elbow/wrist/hand, lower back, pelvis/hip/groin, thigh, knee, and lower leg/ankle/foot. The anatomical areas were chosen based on previous publications describing injury occurrence in ice hockey players [21]. If players indicated an injury in one of these areas (Yes/No), they were provided with the Oslo Sports Trauma Research Centre Overuse Injury Questionnaire (OSTRC-O). OSTRC-O was developed to register overuse injuries [22] but can be applied on both acute and overuse injuries. The questionnaire measures the (1) impact an injury has on a player’s ability to train and play games, (2) potential reductions in training volume and (3) performance due to the injury as well as (4) pain during sport participation. OSTRC-O was modified by asking players to report injuries occurring during the past season. Players then answered some follow-up questions regarding onset of the injury and duration of potential time loss from ice hockey (total inability to participate in training or match, measured in weeks).

### Data management

The burden of each injury was calculated based on the four OSTRC-O items response alternatives, as a severity score [22] ranging from 0 (full participation without health problem) to 100 (highest impact; no participation). Participants who reported no injury/problem were rated with score 0. A total severity score for all injuries during previous season was computed by summarising the severity score for each anatomical area. Injuries characterized by at least one of the following three OSTRC-O responses were defined as “substantial injuries”: (1) moderate or severe reduction in training volume; (2) moderate or severe affected performance; and (3) inability to participate. Injuries characterized by a score of more than 0 in at least one of the 4 OSTRC-O questions without fulfilling criteria to be substantial were defined as “non-substantial injuries”. Players were categorised into either “No”, “Non-substantial” (any injury, but non-substantial) or “Substantial” injury during previous season. SISQ responses were also categorized into low (response options: “not at all stressed” and “just a little stressed”) medium (response option: “stressed to some extent”) and high (response options: “quite stressed” and “very much stressed”).

### Statistical analysis

Spearman correlation was applied for analyses of association between the injury severity score and perceived stress level (PSS) during previous season and current stress level (SISQ) in total group and stratified by sex. The non-parametric correlation was chosen due to skewed distribution of total severity sum of injuries during previous season, and the ordinal five categorical response scale for SISQ. Comparisons of perceived stress during previous season (PSS score) were compared with independent samples t-test between female and male players, and with Analysis of Variance (ANOVA) between injury groups (no, non-substantial, or severe injury) during previous season. The parametric test was used since the PSS score showed a normal/symmetric distribution. Current stress level was compared between sex with Kendall’s tau-B test for the 3 categorical SISQ outcome. For the full Likert scale of current stress level (SISQ), the comparison was performed with Mann-Whitney U test between sex and Kruskal Wallis between injury groups (no, non-substantial or substantial injury during previous season). The non-parametric tests were used due to the single ordinal/Likert scale which was not normally distributed. If omnibus ANOVA or Kruskal Wallis test showed significance, post hoc pairwise comparisons (LSD/Dunn, not Bonferroni-adjusted) were performed. Comparison of stress levels (PSS and SISQ full Likert scale) between injury groups were also performed stratified by sex. All analyses were performed in IBM Statistical Package for Social Sciences SPSS (version 27).

## Results

Among 207 invited female players, 170 (82%) responded to the survey. One hundred ninety of the 623 invited male players (30%) responded to the survey [SHL: 116 (35%); HA: 74 (26%)]. Player characteristics are summarized in Table 1.

**Table 1:** Player characteristics

	**Female players (*n* = 170)**	**Male players (*n* = 190)**
Age Mean (SD) (*n* = 133)	22.9 (4.4)	---^a^
Years playing ice hockey		
Median (IQR; min/max)	17 (14–20; 6–30)	20 (16–24; 9–31)
Years of playing ice hockey on current level		
Median (IQR; min/max)	3 (1–7.5; 0–19)	5 (2–9; 0–22)
Playing position % (n)^b^		
Goaltender	12.4 (21)	11.1 (21)
Defender	36.1 (61)	37.9 (72)
Center	19.5 (33)	22.6 (43)
Forward	39.1 (66)	34.7 (66)
Players reporting injuries during the past season		
No injury % (n)	17.1 (29)	22.1 (42)
Non-substantial injury (only) % (n)	35.3 (60)	33.7 (64)
Substantial injury % (n)	47.6 (81)	44.2 (84)

---^a^Mean age (SD) of male players in the 2022/2023 season: SHL = 25.8 (5.7); HA = 24.7 (4.5)

^b^More than one option possible

### General conditions for playing ice hockey

One-third of female players reported other occupations besides playing ice hockey while almost all male players reported ice hockey as their primary occupation. None of the female players reported that they live with children while 25% of male players reported living with children. Male players living with children did not report absence from ice hockey due to parental leave. Female players reported significantly less access to medical support during games, practices, and in general. The general conditions for playing ice hockey among our sample of female and male ice hockey players is presented in Table 2.

**Table 2:** General conditions for playing ice hockey among female and male players

	**Female players** **% (n)**	**Male players** **% (n)**	***P*-value**
**Everyday conditions**			
Hockey as main occupation	66.9 (113)	98.4 (187)	< .001
Other occupations			
None	24.1 (41)	87.9 (167)	
Work	47.6 (81)	4.7 (9)	
Study	31.2 (53)	7.4 (14)	
Living condition^a^			
Alone	51.8 (88)	23.7 (45)	< .001
With partner	11.8 (20)	3.7 (7)	.004
With parents	18.8 (32)	68.9 (131)	< .001
With kids	0 (0)	25.3 (48)	< .001
With another person	20 (34)	0.5 (1)	< .001
**Medical support**			
Support during games			< .001
Always	56.5 (96)	98.4 (187)	
Sometimes	24.1 (41)	1.6 (3)	
Never	4.1 (7)	0 (0)	
Don’t know	15.3 (26)	0 (0)	
Support during practice			< .001
Always	26.5 (45)	70.5 (134)	
Sometimes	35.3 (60)	24.7 (47)	
Never	28.2 (48)	4.7 (9)	
Don’t know	10 (17)	0	
Medical profession attendant^b^			
Physician	35.7 (50)	84.4 (157)	< .001
Physio	76.4 (107)	85.5 (159)	.037
Naprapath	5 (7)	30.1 (56)	< .001
Nurse	15 (21)	6.5 (12)	.011
Chiropractor	0.7 (1)	7 (13)	.006
Other	14.3 (20)	8.1 (15)	.072
Med contact to turn to (Yes)	81.1 (137)	97.4 (185)	< .001

^a^More than one response option possible

^b^Among athletes with any medical support reported. More than one response option possible

### Perceived stress among female and male players

**Fig. 1:**
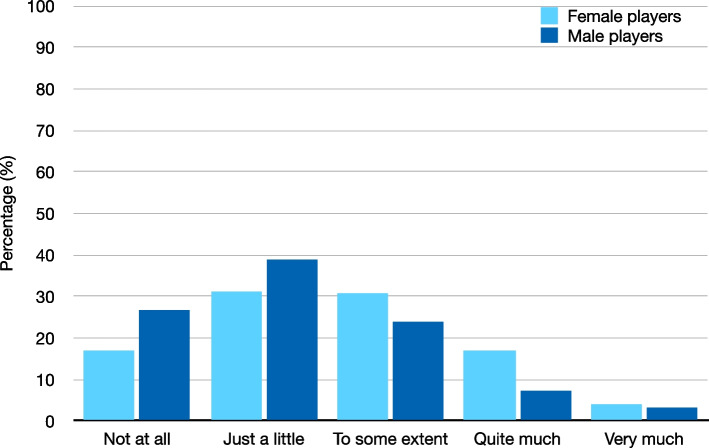
Current stress levels in female and male players according to the Single Item Stress Question

**Fig. 2:**
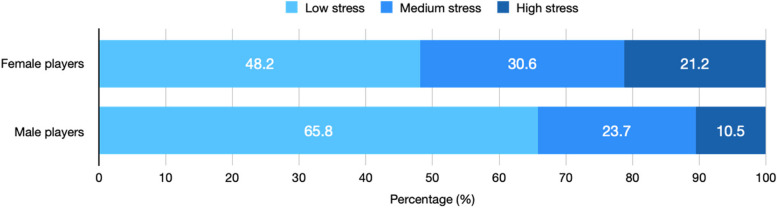
Percentage of female and male players levels reporting low, medium, and high levels of stress according to the Single Item Stress Question

Female players perceived higher stress levels during the previous season compared to their male counterparts [Mean PSS score (SD): 17.4 (5.6) vs 14.1 (5.6); mean difference (95% CI): 3.3 (2.1–4.5), Cohen’s D (95% CI): 0.6 (0.4–0.8); *p* < 0.001]. Female players also perceived significantly higher levels of current stress (SISQ) than male players [Median (IQR): 3 (2–3) vs 2 (1–3); *p* < 0.001]. Distribution of players in all SISQ Likert response categories is illustrated in Fig. 1. There was also an association between sex and the 3-categorical SISQ (*p* = 0.001), with higher levels of stress among female players (Fig. 2).

### Association between stress and injuries during previous season

There was a weak but statistically significant positive association between total injury severity score during previous season and perceived stress level during the previous season and current stress level. The observed correlations were stronger among male players than among female players (Table 3).

**Table 3:** Correlation between stress and severity score of injuries during previous season

	Total Rho	Female players Rho	Male players Rho
PSS sum (previous season)	0.29**	0.16*	0.38**
SISQ (currently)	0.24**	0.16*	0.29**

*PSS* Perceived Stress Scale, *SISQ* Single Item Stress Question

*P < .05; **P < .001

Perceived stress levels (PSS) during the previous season differed significantly between players that reported to have had no, non-substantial, or substantial injuries during the previous season (*p* < 0.001). Post-hoc testing showed that players with substantial injury had significantly higher stress levels than players without injury (*p* < 0.001), and players with non-substantial injuries (*p* < 0.001), while the difference between players with no injury and with non-substantial injuries was not statistically significant (*p* = 0.152). Sex stratified analyses showed that these significant differences were present among men but not among women (Table 4).

**Table 4:** Perceived stress levels during the previous hockey season among players reporting no injury, non-substantial injuries, or substantial injuries during the same season

	Total (*n* = 360)		Females (*n* = 170)		Males (*n* = 190)	
	PSS	Difference	PSS	Difference	PSS	Difference
	Mean (95%CI)	Mean (95%CI)	Mean (95%CI)	Mean (95%CI)	Mean (95%CI)	Mean (95%CI)
No injury	13.4 (12.1; 14.7)^c^	*Ref*	16.5 (14.5; 18.5)	*Ref*	11.3 (9.7; 12.9)^c^	*Ref*
Non-substantial injuries	14.6 (13.6; 15.6)^c^	1.2 (-0.4; 2.8)	16.5 (15.1; 17.9)	0.03 (-2.4; 2.5)	12.8 (11.5; 14.1)^c^	1.5 (-0.5; 3.6)
Substantial injuries	17.4 (16.5; 18.2)^ab^	4.0 (2.4; 5.5)**	18.3 (17.1; 19.6)	1.9 (-0.5; 4.2)	16.4 (15.3–17.5)^ab^	5.2 (3.2; 7.1)**

*PSS* Percieved stress scale (0-40)

^**^P < .001 (reference No injury)

^abc^Statistically significant difference compared with a = No injury, b = Non-substantial injury, c = Substantial injury

**Fig. 3:**
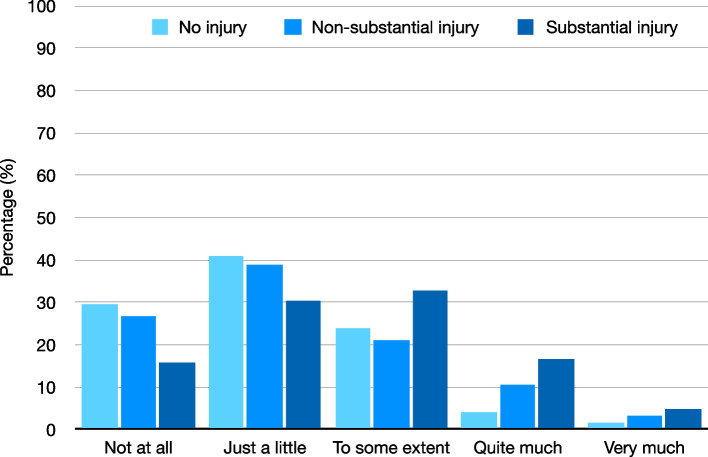
Current stress levels in players with no, non-substantial, or substantial injury during the previous season according to the Single Item Stress Question

Current stress levels (SISQ, Fig. 3) also differed significantly between players that reported no injury, non-substantial injury, or substantial injuries during the previous season (*p* < 0.001). Post-hoc testing revealed these differences to be non-significant between players without and with non-substantial injury (*p* = 0.315). Players who reported substantial injuries during the previous season differed statistically significant from players that reported no injuries (*p* < 0.001), and non-substantial injuries (*p* = 0.001). Sex stratified analyses showed that these significant differences were present among men (*p* < 0.001) but not among women.

## Discussion

Our study is the first of its kind to describe and compare general conditions for playing ice hockey, perceived stress, and the association between stress and past seasons injury in elite female and male ice hockey players. General levels of perceived stress were higher among female players, who reported significantly less access to medical support in addition to a higher burden of work or studies outside of hockey as compared to their male counterparts.. We found significant but weak correlations between both perceived past season- and current stress level and past season’s injury severity score for the total group, though this correlation was stronger in male players. Stress levels were higher in players with substantial injury than players without injuries during the previous season. These differences were statistically significant only among males.

### General conditions for playing ice hockey

Female players reported significantly less access to medical support than male players. Only around half of all females reported the presence of medical support during games and only one in four reported a constant medical presence during practices. Ice hockey is a high-speed collision sport and reported to be among the sports with highest injury incidence and risk for permanent medical impairment here in Sweden [23]. Therefore, the reported lack of medical support on the highest national level for females is concerning while the high level of support among male players further alludes to a vast contrast in the distribution of resources between sexes. The unequal distribution of resources between sexes is further illustrated by the fact that only one in four female players reported playing ice hockey to be the only occupation while nine out of 10 male players report ice hockey to be their only occupation. Although level of financial compensation for playing ice hockey was not included in our survey, it is likely that economy is the driver for competing occupations among females. According to the Global Gender Gap Report [24], Sweden is the top-performing country in terms of closing the gap between income of men and women with 18% left to full closure. However, the economic gap appears to be much larger in elite ice hockey than in the general population. In a 2020 survey by a Swedish employer union and the Swedish Ice hockey player union, it was reported that a male elite player has a mean salary corresponding to that of a whole team in the highest league for females [25]. That means that very few female ice hockey players can make a living from playing their sport in Sweden, a fact that the SWHL aims to change by reaching a bench mark of 25% of all league players living from playing ice hockey by 2025 [26]. The different conditions to support oneself may also reflect family planning. In our study one in four male players report to have children while only one of all 170 female players reported to have children. The average age among our female sample is relatively young, but conditions may affect future planning and development. In a previous survey-study, female players reported that they refrain from having children because of their hockey career and 40% reported to cease playing once they have the desire to build a family [25]. The former NHL coach Fred Shero once said: “Hockey is where we live. Life is just a place where we spend time between games” [27]. Our results indicate that the conditions to play ice hockey and therefore also the time spend between games is very different for females and males in hockey. In 2008, female ice hockey players in a qualitative study reflected upon the unequal distribution of material resources such as ice time and economic support among women and men and came to the conclusion that ice hockey never can be more than a hobby to them [1]. Today, 15 years later we start to see changes that can alter this reality, but we still have a long way to go. One of the questions we aimed to answer in this study is whether potentially different playing conditions may relate to perceived stress among female and male players. With the above described differences in general conditions one could expected that female players experience more stress due to the occupational demands placed on them which both may affect the demand-control balance, and also affect the time for recovery; which is an important aspect to avoid unhealthy consequences from stress [10, 28].

### Perceived stress levels

As one would expect from the rational provided in the paragraph above, we found higher levels of perceived stress among female players than among male players. While this difference may be explained by the different general conditions for being an elite ice hockey player, women normally report higher levels of stress even in the general population [17]. Compared to normative data, the difference in perceived stress among female and male players corresponds to the observed difference between genders in the general population (18–34 years of age) [17]. The comparison to the general population also indicates that ice hockey players report similar levels of perceived stress as the general population [17]. The next question we wanted to answer was whether stress levels would be associated to the presence of injury in the previous season.

### Association between perceived stress and injuries during previous season

We found statistically significant but weak correlations between perceived stress (current and during the past season) and past season’s injury severity score. Previous research indicates a bidirectional correlation between perceived stress and injury as stress correlates to injury risk and injured athletes experience negative stress responses [12–14]. We found stronger associations between stress and injuries in male players, which could also be explained by the differences in general conditions we found. In male players, playing hockey is their main occupation and an injury therefore posts a considerable threat to their career and economic security. The strength with which an athlete identifies as an athlete is called athlete identity [29]. Strong athletic identity is considered a risk factor for mental health problems following injury [30]. The threat of losing that identity due to injury and psychological distress related to injury is associated with retirement from sport and may explain why the correlation between perceived stress and injury was stronger among male than among female players. We saw a gradually increasing difference in stress levels when we compared players without injury to those with non-substantial and substantial injuries. However, these differences were only significant for male players without and with substantial injuries. Since substantial injuries are characterized by a moderate to severe reduction in training volume and ability to perform or total inability to participate in ice hockey, the argument regarding more stress for those who have more to lose receives further support.

### Methodological considerations

A strength of our study is that we investigate a topic that has not been investigated earlier. To our knowledge this is the first peer-reviewed description of sex-differences in conditions for playing ice hockey on the highest national level. With regard to the general underrepresentation of female athletes in sports medicine research [31], our study can be considered an important contribution to the field. It must be acknowledged that we have not covered all potentially relevant aspects of general conditions for athletes with the survey we developed for this current study and that we did not perform any formal pilot testing of the survey. However, in the absence of comparable studies there are no validated instruments to measure what we aimed to describe. The instrument we used to describe retrospective occurring injuries [22] is usually used in prospective studies and had to be modified for the intended use in this study. Nevertheless, similar modifications of the instrument have been used in studies with comparable design [32] and even though retrospective recall of injury occurrence is not optimal it can be considered a valid recording method [33]. Unfortunately, players’ age was not collected in the survey for male players. Therefore, we report mean age for the underlying population in respective leagues (SHL and HA) and we do not assume that our sample deviates considerably from this. The high response rate for female players indicates good generalizability of our results, while the much lower response rate among male players calls for caution. However, as the stress levels observed in our studies are comparable to normative data [17] and the injury data is comparable to previous studies [34], we do not believe that our results are affected by such a selection bias. It also must be acknowledged that the cross-sectional design of our study does not permit us to make causal interferences between injuries and perceived stress. As highlighted by previous research, perceived stress may lead to athletic injuries, but athletic injuries may also lead to perceived stress [12–14]. Prospective studies are needed to further explore the causal direction of this interaction.

## Conclusions

General conditions for playing ice hockey are unequal for female and male athletes. Female players report higher stress levels that may be due to the different sporting conditions although perceived stress levels are generally higher in females in the general population. We found associations between perceived stress and injury occurrence as well as severity during the past season. These associations were stronger among male players than female players, which may be due to greater economic dependency.

## Data Availability

The datasets used and/or analysed during the current study are available from the corresponding author on reasonable request.
